# FTO promotes colorectal cancer progression and chemotherapy resistance via demethylating G6PD/PARP1

**DOI:** 10.1002/ctm2.772

**Published:** 2022-03-16

**Authors:** Jiyan Wang, Yaya Qiao, Mingming Sun, Huanran Sun, Fei Xie, Hongkai Chang, Yingzhi Wang, Jiaqi Song, Sizhen Lai, Chenxin Yang, Xichuan Li, Shuangping Liu, Xuanzhu Zhao, Kemin Ni, Kewei Meng, Shuai Zhang, Changliang Shan, Chunze Zhang

**Affiliations:** ^1^ State Key Laboratory of Medicinal Chemical Biology, College of Pharmacy and Tianjin Key Laboratory of Molecular Drug Research Nankai University Tianjin China; ^2^ School of Integrative Medicine Tianjin University of Traditional Chinese Medicine Tianjin China; ^3^ Tianjin Key Laboratory of Animal and Plant Resistance, College of Life Sciences Tianjin Normal University Tianjin China; ^4^ Department of Pathology, Medical School Dalian University Dalian Liaoning China; ^5^ School of Medicine Nankai University Tianjin China; ^6^ The department of gastrointestinal surgery, Tianjin First Central Hospital Nankai University Tianjin China; ^7^ Department of Colorectal Surgery Tianjin Union Medical Center Tianjin China; ^8^ Tianjin Institute of Coloproctology Tianjin China; ^9^ The Institute of Translational Medicine Tianjin Union Medical Center of Nankai University Tianjin China

Dear Editor,

Cytotoxic chemotherapy has long been the backbone of treatment for colorectal cancer (CRC) in patients. In spite of advances in therapy, the 5‐year survival rate is still unsatisfactory, mostly due to chemotherapy resistance in the therapy process.^1^, ^2^ In our study, we demonstrated that the fat mass and obesity‐associated (FTO) protein promotes CRC progression and increases chemotherapy resistance, thus, targeting FTO is a promising strategy for therapy CRC, which not only blocks tumor growth, but also reverses chemotherapy resistance.

The m6A modification is a dynamically reversible process, added by methyltransferases (Writers: METTL3, METTL14) and removed by demethylases (Erasers: FTO, ALKBH5). To explore the fundamental role of m6A modification during chemotherapy, we found that the level of m6A modification was decreased in the cells treated with 5‐FU and cisplatin, the clinical treatment drugs for colorectal cancer (Figure 1A). Later, we also screened out FTO as the major driver in regulating m6A modification during 5‐FU and cisplatin treatment from m6A methyltransferases and demethylases (Figure 1B–D; Figure S1A‐C). What's more, we proved that FTO's response to chemotherapeutic drugs depends on its m6A demethylase activity (Figure 1E). Lastly, knockdown of FTO also increased the sensitivity of CRC cells to chemotherapy drugs (Figure 1F; Figure S1D‐E). In short, FTO, as a demethylase, responses to chemotherapeutic drugs stimulation is universal. These finding means that FTO plays an antagonistic role in therapizing CRC by chemotherapeutic drug.

**FIGURE 1:**
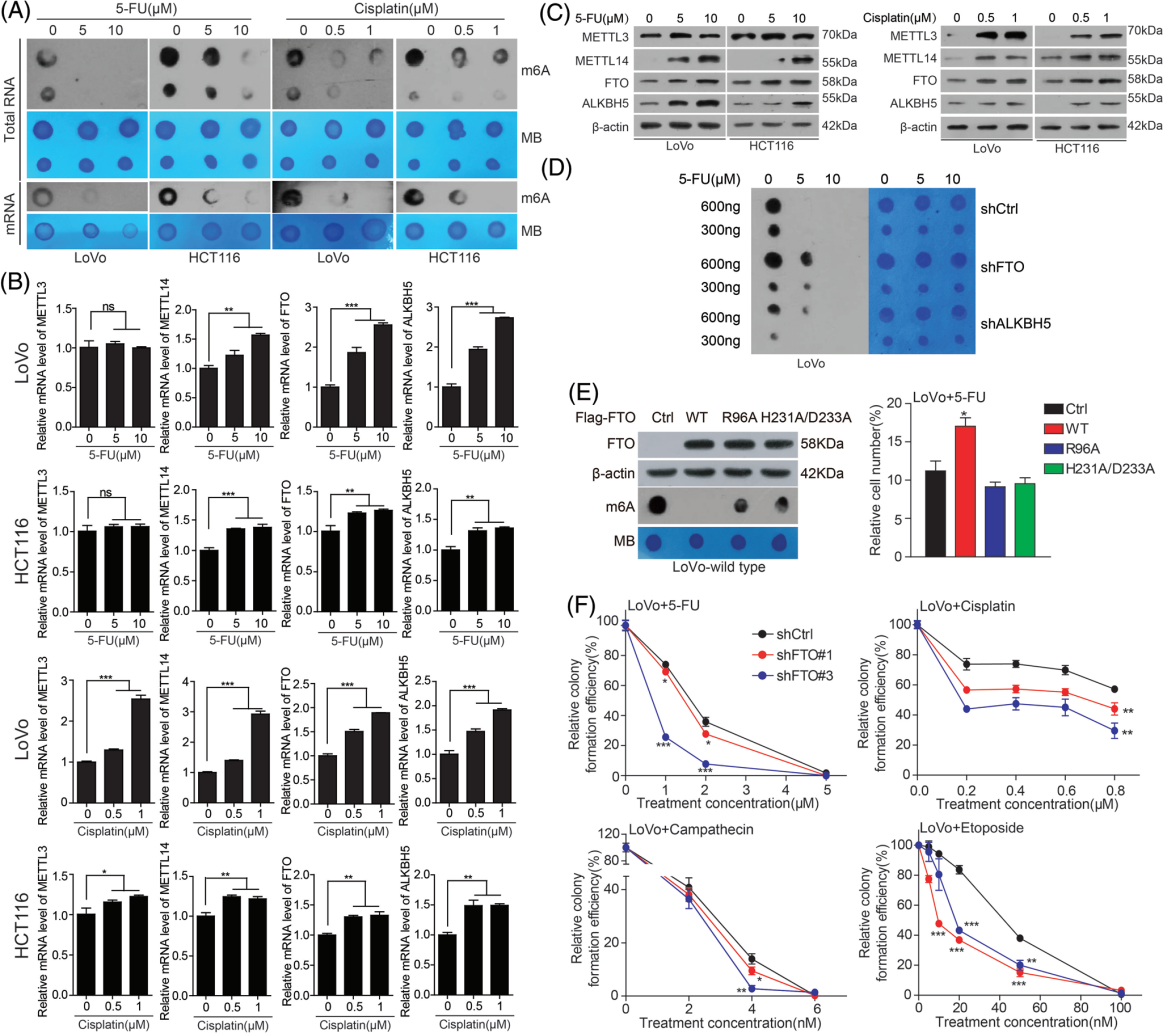
FTO mediates chemotherapy process in CRC. (A‐C) The m6A modification level of total RNA and mRNA and expression of m6A regulator with treatment of 5‐FU and cisplatin. (D) The m6A modification level in FTO/ALKBH5 knockdown cells with treatment of 5‐FU. (E) The sensitivity of 5‐FU was detected in LoVo cells, which overexpressed FTO wild ‐type or mutant. (F) The sensitivity of **5‐FU and cisplatin** in FTO knockdown cells. Data are presented as the means ± SD (n = 3) *p < 0.05, **p < 0.01, ***p < 0.001

To explore the role of FTO during chemotherapy drugs treatment, we found that 5‐FU and cisplatin treatment increased ROS (Figure 2A), which are consistent with previous studies.^3^ At same time, knockdown of FTO also induced ROS levels (Figure 2B). As NADPH plays a vital role in maintaining ROS, this will post damage to genome stability and cell senescence. Indeed, the loss of FTO reduced NADPH/NADP^+^ ratio, not NADH/NAD^+^, (Figure 2C and D). Besides, targeting FTO also disrupt DNA damage repair and ultimately promotes cell senescence (Figure 2E–G). Interestingly, cell senescence and DNA damage were restored by eliminating ROS (Figure 2H and I), which proved that FTO regulates CRC cell senescence and DNA damage by regulating ROS (Figure 2J). To explore whether FTO regulates these processes dependent on its activity, we treated cells with FTO inhibitors (Rhein/FB23‐2) and found that the inhibition of FTO activity also induced ROS, DNA damage, and cell senescence (Figure S2). These results suggested that targeting FTO to therapy CRC as the same function as chemotherapeutics (5‐FU and cisplatin), which induce ROS and break genome stability. More interesting, we found that targeting FTO by shRNA or inhibitor decreased CRC cell proliferation and tumor growth in vitro and in vivo (Figure S3).

**FIGURE 2:**
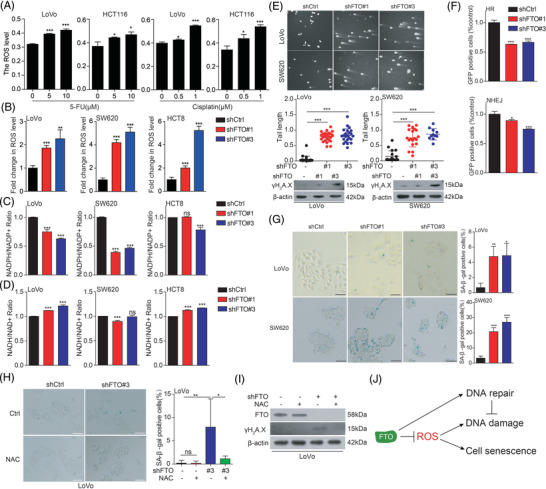
FTO regulates ROS level, DNA damage repair in CRC. (A) The ROS level of CRC cells with treatment of 5‐FU and cisplatin. (B‐G) The effect of knockdown of FTO on ROS, DNA damage repair and cell senescence. (H,I) The cell senescence and DNA damage were determined in CRC cells stable knockdown FTO treated with NAC. (J) A schematic model illustrating our findings on FTO‐mediated DNA damage repair and cell senescence was shown. Data are presented as the means ± SD. *p < 0.05, **p < 0.01, ***p < 0.001

G6PD, as first key enzyme of pentose phosphate pathway (PPP), is main producer of NADPH.^4^ PARP1 plays an indispensable role in DNA damage repair, especially DNA double‐strand breaks (DSB). Thus, to explore whether FTO regulates redox homeostasis and DNA repair process are mediated by G6PD/PARP1 through m6A modification; First, we performed real‐time PCR and western blotting assay and found that FTO regulates the mRNA and protein levels of G6PD and PARP1 (Figure 3A,B; Figure S4A–C). Next, we found that FTO removes the m6A modification on *G6PD/PARP1* mRNA by MeRIP‐seq/MeRIP‐qPCR assay (Figure 3C; Figure S4D–F). As the m6A modification is recognized and bound by m6A‐binding proteins (Readers), which control mRNA fate and function. Lastly, we identified YTHDF2 as G6PD/PARP1 m6A reader protein (Figure 3D), which mediates the degradation of mRNA.^5^ Indeed, we found that the *G6PD/PARP1* mRNA stability was markedly decreased upon FTO knockdown (Figure 3E), while, the decreased *G6PD/PARP1* mRNA stability was restored by knockdown of YTHDF2 in FTO depletion cells (Figure 3F; Figure S4G). What's more, we identified specific m6A modification sites on *G6PD/PAPR1* mRNA (Figure 3G and H), and confirmed that the YTHDF2 binding ability on *G6PD/PAPR1* mRNA was decreased when these sites are mutated (Figure 3I).

**FIGURE 3:**
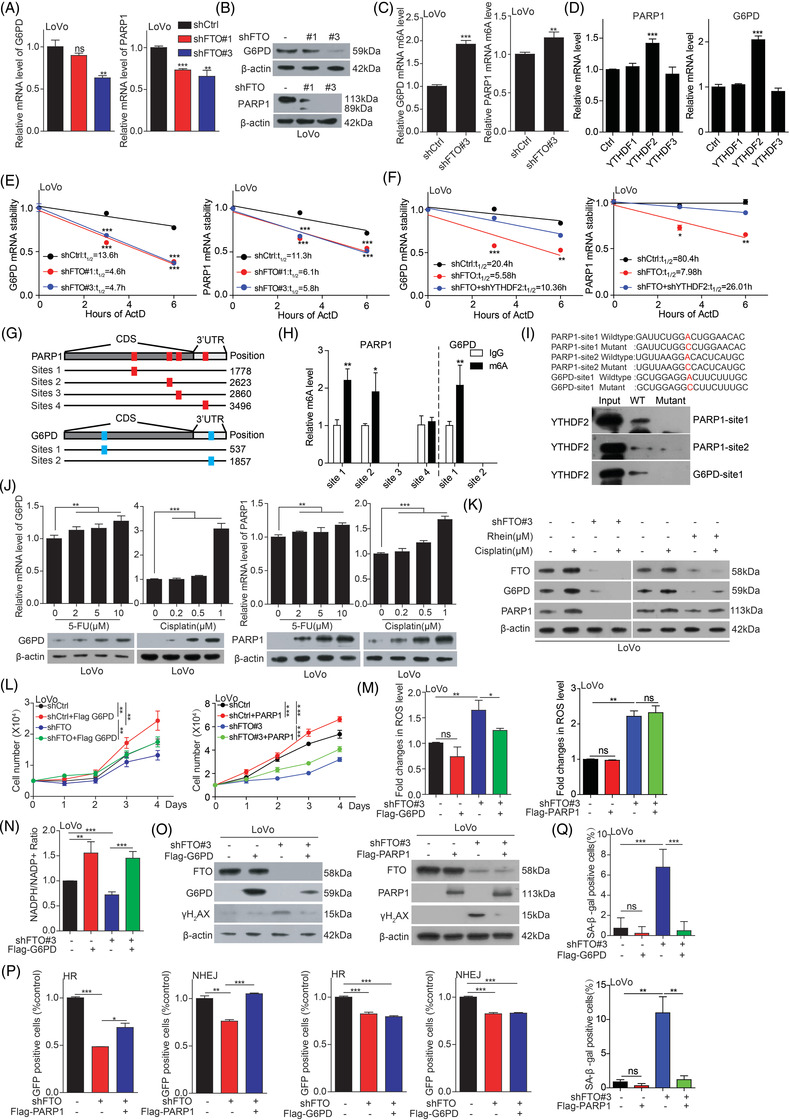
FTO regulates *G6PD/PARP1* mRNA stability in an YTHDF2 dependent manner. (A‐B) The expression of G6PD/PARP1 in FTO knockdown cells. (C) The m6A level of *G6PD/PARP1* mRNA in FTO knockdown cells. (D) The screening of reader protein binding *G6PD/PARP1* mRNA. (E,F) The mRNA stability of G6PD/PARP1 in FTO knockdown cells with or without knockdown of YTHDF2. (G‐I) The specific binding site of YTHDF2 and G6PD/PARP1 mRNA. (J) The expression of G6PD/PARP1 in CRC cells with treatment of 5‐FU or cisplatin. (K) The expression of FTO, G6PD and PARP1 in knockdown or inhibition of FTO cells with or without cisplatin treatment. (L‐Q) The cell growth, ROS level, NADPH level, DNA damage, HR or NHEJ efficiency and cell senescence in FTO knockdown cells with or without overexpression of PARP1. Data are presented as the means ± SD *p < 0.05, **p < 0.01, ***p < 0.001

To determine whether G6PD/PARP1 is involved in regulating chemotherapy response mediated by FTO, we treated CRC cells with 5‐FU and cisplatin and found that both G6PD and PARP1 mRNA and protein levels were highly increased in 5‐FU or cisplatin treated CRC cells (Figure 3J; Figure S4H,I), while the increased G6PD and PARP1 were blocked by the inhibition or knockdown of FTO (Figure 3K). These results confirmed that G6PD/PARP1 as the key downstream involving in regulating CRC chemotherapy resistance mediated by FTO. To explore the role of G6PD/PARP1 in regulating oxidative stress and DNA damage, we forced expression of G6PD/PARP1 in FTO knockdown cells. The results showed that overexpression of G6PD, not PARP1, antagonized the increased ROS level; and exogenous of PARP1, not G6PD, rescued the decreased HR and NHEJ in FTO knockdown cells. Finally, the cell proliferation, DNA damage, and cell senescence were restored not only by overexpression of G6PD, but also by overexpression of PARP1 (Figure 3L‐Q; Figure S4J–L). These results suggest that G6PD mediates the balance of NADPH and ROS to affect DNA damage, while PARP1 mediates the process of DNA damage repair, and the two pathways are both linked and independent of each other.

Based on our findings that FTO promotes the progress of CRC mediated by G6PD and PARP1, we proceeded to explore the clinical relevance between G6PD/PARP1 and FTO. In CRC patient samples (Cohort 1 and 2) and CRC cells, G6PD/PARP1 expression positively correlated with FTO in CRC tissues, and negatively correlation with the m6A level (Figure S5A‐O; Table S1‐S6). In addition to 5‐FU and cisplatin, Olaparib is also a chemotherapy drug for the treatment of colorectal cancer.^6^ The knockdown or inhibition of FTO also increased the sensitivity of CRC cells to Olaparib (Figure 4A,B; Figure S5P‐Q). In CRC‐bearing xenograft mouse model, the Rhein/Olaparib combination therapy significantly restrained tumor growth compared to control group (Figure 4C‐I; Figure S5R,S). In summary, these results suggested that the critical role of FTO in promoting CRC progression and FTO has potential as target for treating CRC.

**FIGURE 4:**
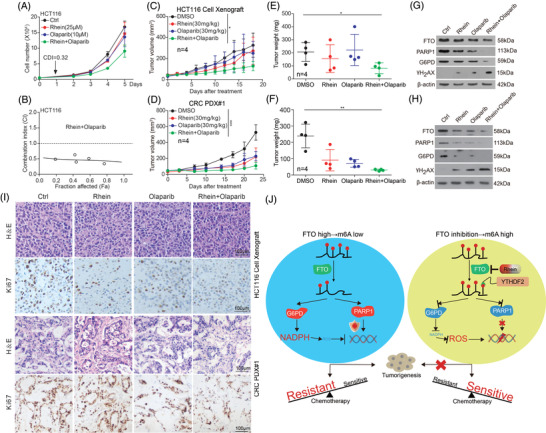
FTO enhanced the anti‐tumor effects of Olaparib in CRC. (A‐I) Inhibition of FTO enhances the sensitivity of CRC to Olaparib in vitro and in vivo. (J) Proposed model: The role and mechanism of FTO in CRC progression. Data are presented as the means ± SD. *p < 0.05, **p < 0.01, ***p < 0.001

Our results demonstrate that targeting FTO significantly suppresses cancer cell growth and enhances chemotherapy sensitivity, which not only mediating the balance of intracellular ROS by regulating G6PD expression, but also maintaining genome instability by regulating PARP1 expression (Figure 4J). This is analogous to throwing a “bomb” (ROS) to induce DNA damage also disabling the “anti‐missile system” (PARP1) to block DNA repair. These findings shed light on new molecular mechanisms of CRC development and treatments mediated by m6A modification and provide new insights into developing effective therapeutic strategies for CRC.

## CONFLICT OF INTEREST

The authors declare no conflict of interest.

## Supporting information

Supporting Information 1
