# Supplementary material for: FTO promotes colorectal cancer progression and chemotherapy resistance via demethylating G6PD/PARP1
Source: Clin Transl Med. 2022 Mar 16;12(3):e772. doi: 10.1002/ctm2.772 (PMC8926902; doi:10.1002/ctm2.772)
Supplement: Supplementary file 1 — Supporting Information 1 [file CTM2-12-e772-s001.pdf]

Supplementary information

Materials and methods

1. Colorectal cancer patient samples (Cohort 1 and Cohort 2)

The frozen colorectal cancer tissues (Cohort 1 = 30) and colorectal cancer tissue microarrays （Cohort 2 = 29） were provided from Tianjin Union Medical Center (Tianjin, China). These tumor tissues included 30 colorectal cancer tissues and 30 distal non-tumor colorectal tissues. These microarrays included 29 colorectal cancer tissues, 29 adjacent non-tumor colorectal tissues and 29 distal non-tumor colorectal tissues. The study protocol was approved by the Institute Research Ethics Committee at Nankai University.

2. Cell lines and cell culture

The colorectal cancer (CRC) cells LoVo, HCT116, HCT8, and SW620 were cultured in RPMI1640 supplemented with 10% fetal bovine serum (FBS). NCM460 was cultured in DMEM supplemented with 10% FBS. HEK293T cells were cultured in DMEM with 10% FBS, antibiotics, and 10 mM HEPES, and used for lenti-virus package. All cells were cultured at 37°C in an incubator supplied with 5% CO_2.

3. Plasmids construction and transfections

We generated METTL3, METTL14, FTO, ALKBH5, and YTHDF2 stable knockdown CRC cells by using lentiviral vectors harboring shRNA constructs (Transheep Biological Corporation, China). To produce lentivirus, we co-transfected each lentiviral shRNA constructs with viral packaging plasmids (psPAX2 and pMD2.G) into HEK293T human embryonic kidney-derived cells using Polyethylenimine (PEI) Transfection Reagent (Polysciences, USA) according to the manufacturer's instructions. Two days after transfection, we collected supernatant medium containing lentivirus and filtered viral supernatant by using 0.45 μm filter, then we infected targeting cells and subjected to puromycin selection.

The overexpression plasmids of pCDNA3.0-Flag-FTO (wildtype, R96A, and H231A/D233A) was kindly provided by Yang Caiguang (Chinese Academy of Sciences, Shanghai). The silent mutation of FTO were cloned into pCDNA3.0-basic by using the Fast Mutagenesis System (TransGen Biotech, China). The plasmid pLVX-Flag-G6PD and pCDNA3.0-Flag-PARP1 were constructed in our laboratory. For transient transfections, cells were grown to 80% confluency and transfected with plasmids using Polyethylenimine (PEI) (Polysciences, USA) according to the manufacturer's protocol. Unless otherwise noted, the overexpression were transient transfections.

4. Western blot

The cells were harvested and lysed with the RIPA lysis buffer at 4°C. The cell lysate was centrifuged and the protein concentration was quantified by Ultra Trace Ultraviolet Spectrophotometer. An equal amount of cell lysate from each sample was loaded to the SDS-PAGE. The proteins were transferred onto PVDF membranes (Millipore, USA), blocked with 5% skim milk and incubated with antibodies against FTO, PARP1, G6PD, $\gamma H_2A.X$, β-actin etc. HRP conjugated Goat Anti-mouse or

Anti-rabbit IgG was used as secondary antibody. The signal was detected by Immobilon Western HRP Kit (Millipore, USA).

5. Cell proliferation assay

CRC cells that were knocked down or treated with inhibitors were seeded in 24-well plates. On second day of seeding, the counting is started and lasts four day.

6. Colony formation assay

CRC cells that were knocked down or treated with inhibitors were seeded in 6-well plates. The cells were continuous cultured for 1-2 weeks. Cells were stained by Crystal violet for counting. Colony number was measured by Image J software.

7. Comet assay

Briefly, cells were embedded into low melting agarose and covered by lysis buffer overnight at 4°C. Grass slides were immersed into TAE buffer and electrophoresis was applied at 20 V for 20 min. DNA was stained with ethidium bromide (EB) and images were acquired under a Leica Fluorescence Microscope.

8. Immunofluorescence

For immunofluorescence, cells were cultured on coverslips for 24 h before experimental treatment. Samples were first fixed with 4% paraformaldehyde (15 min), rinsed three times with PBS, permeabilized with 0.1%Triton X-100 in PBS (20 min) and then blocked with 2% BSA in PBS (60 min). The samples were incubated with indicated primary antibodies made up in 2% BSA in PBS overnight at 4°C and rinsed three times with PBS prior to 30 min incubation with secondary antibodies conjugated to Alexa Fluor-555. After being washed three times with PBS, the coverslips were mounted using DAPI Fluoromount-G. Fluorescent micrographs were obtained using laser scanning confocal microscopy (Leica, Germany).

9. SA-β-gal staining assay

SA-β-gal staining kit was used to stain senescent cells. According to the manufacturer's instructions, cells were fixed for 15 min, stained for 1 day and analyzed. Senescent cells, identified as blue-stained cells, were captured with light microscopy.

10. Measurement of intracellular ROS

Intracellular ROS was measured by the fluorescence probe DCFDA following the manufacturer's instructions. In brief, cells were seeded in 6-well plates. Twenty-four hours later, cells were rinsed with PBS and loaded with 5 μM carboxy-H_2DCFDA for 30 min in 37°C. The cells were then harvested, re-suspended in PBS and analyzed using a microplate reader (Thermo) at 488/525 nm.

11. Measurement of NADPH and NADH

Intracellular NADPH levels were assayed using $NADP^+$/NADPH assay kit according to the manufacturer's instructions. Briefly, cells were washed with cold PBS and pelleted. Homogenized samples were treated with $NADP^+$/NADPH extraction buffer (200 μL), and of which extraction buffer (100 μL) to evaluate $NADP^+$ and NADPH levels. The additional extracts (100 μL) were heated at 60°C for 30 min to evaluate NADPH levels. Finally, assay buffer (100 μL) and develop buffer (10 μL) were added to incubate the extracts. $NADP^+$ or NADPH was measured in the supernatant by reading the fluorescence at 450 nm. Similarly, NADH level was measured by a

NAD^+/NADH Assay Kit.

12. HR/NHEJ reporter assay

Briefly, U2OS-DR-GFP and U2OS-EJ5-GFP cells were infected with lentivirus shFTO. The following day, the I-SceI expression vector was transfected using Polyfectine (PEI) and cells were further grown for two days. To assess HR and NHEJ frequency, the GFP positive cell population was quantified with a FACS Calibur flow cytometer (BD, USA).

13. Real-time quantitative PCR (RT-qPCR)

Total RNA isolated with the TRIzol reagent was subjected to reverse transcription using the PrimeScript RT reagent Kit with gDNA Eraser (RR047A, Takara). RT-qPCR reactions were performed with the TB Green™ Premix Ex Taq™ II (RR820A, Takara) and primers listed in Table S7. Gene expression was calculated using the comparative $2^{-\Delta\Delta CT}$ method with the actin for normalization.

14. mRNA stability assay

Actinomycin D (5 mg/mL) was treated to cells for the indicated times, and the mRNA levels at each time point were analyzed by qPCR.

15. m6A dot blot assay

Dot blot assay was performed to determine the global m6A abundance of total RNA or mRNA. The mRNA was enriched with PolyATract mRNA isolation System IV (Z5310, Promega) in accordance with the manufacturer's instructions. In brief, total RNA or mRNA was mixed with SSC buffer and denatured at 65°C for 5 min. Then, the RNA samples were loaded on the Amersham Hybond-N+ membrane (RPN119B, GE Healthcare), and crosslinked to the membrane by UV. The membrane was stained by methylene blue for control. Then, the membrane was blocked with 5% skim milk and incubated with m6A antibody overnight at 4°C. After rinsed with PBST, the membrane was incubated with secondary antibody for 1 h. The signal was detected by Immobilon Western HRP Kit (Millipore, USA).

16. m6A methylated RNA immunoprecipitation-qPCR (MeRIP-qPCR)

Briefly, total RNA was isolated and an equal amount of RNA incubated with m6A antibody or normal rabbit IgG mixed Protein A/G Beads (Santa Cruz, CA) in 500 µL buffer containing 40U RNase inhibitors overnight at 4°C. RNA with m6A modifications were immunoprecipitated by m6A antibody-conjugated beads, washed three times and incubated with proteinase K digestion buffer. RNA was finally purified by Trizol/chloroform extraction and analyzed by RT-qPCR. For identification of specific m6A sites, we used 10× fragmentation buffer (mix 800 µL of molecular biology-grade, RNase-free water with 100 µL (1 M stock) of Tris-HCl (pH 7.0) and 100 µL (1 M stock) of $ZnCl_2$. Freshly prepare the buffer. The final concentrations in the buffer concentrate are 100 mM Tris-HCl and 100 mM $ZnCl_2$) to fragment the RNA prior to incubation. The qPCR primers need to be designed on both sides of the predicted m6A site and listed in Table S7.

17. RNA immunoprecipitation (RIP) assay

Briefly, cells transfected with Flag-YTHDF1/YTHDF2/YTHDF3 were harvested, lysed and incubated with Flag-beads overnight at 4°C. Then washed the beads three times and incubated with proteinase K to digest the protein. Chloroform-isopropanol

reagent was used to extract the RNA in the immunoprecipitates and inputs. The RT-qPCR was conducted to quantify G6PD/PARP1 mRNA.

18. RNA pulldown assay

Biotin-labelled RNA oligonucleotides were synthesized by the GENEWIZ. Briefly, the single-stranded RNA baits were denatured at 99°C for 10 min and put on ice immediately. Equal RNA oligonucleotides of each was added to 50 μL streptavidin magnetic beads (Thermo Fisher Scientific) in PBS buffer at 4°C for 4 h. RNA bait-conjugated streptavidin beads were then incubated with HCT116 cells extract in lysis buffer overnight at 4°C. After extensive washing, RNA-protein complexes were dissolved in 1× SDS buffer.

19. Methylated RNA immunoprecipitation sequencing (MeRIP-seq)

The LoVo-shCtrl and LoVo-shFTO#3 cells were collected and resuspended with TRIzol, and subjected to MeRIP-seq and RNA-seq using sequencing platform (BGI, Wuhan, China). The m6A peaks reads coverage were showed by IGV software.

20. Immunohistochemistry

Immunohistochemistry was performed on paraffin-embedded sections. Tissue sections were dewaxed and rehydrated using standard protocol. Antigen retrieval was performed by boiling samples in citrate buffer for 15 min. Endogenous peroxidase activity was inhibited by using 3% hydrogen peroxidase. Sections were blocked in 3% BSA in PBS and incubated in primary antibody overnight at 4°C. Sections were rinsed in PBS and developed using DAB. Sections were counterstained with hematoxylin. Quantification of staining was performed using Image J software.

21. Evaluation of IHC staining

All specimens were examined by two investigators (JYW & YYQ) who did not possess knowledge of the clinical data. Briefly, the IHC staining for FTO or PARP1 or G6PD was semi-quantitatively scored as '-' (negative, no or less than 5% positive cells), '+' (5-25% positive cells), and '++' (more than 25% positive cells, considered as strongly positive).

22. Cell-derived tumor xenograft (CDX) and patient-derived tumor xenograft (PDX)

For CDX model, nude mice (female, 4-6-week-old) were subcutaneously injected with 5×10^6 HCT116 cells on the both flank. For PDX model, the fresh CRC tumor tissues used to establish the PDX mice model were provided from Tianjin Union Medical Center (Tianjin, China). The patient tumors were divided into small pieces and then inoculated on both flank of nude mice. After the tumor grows to a suitable size, the tumor was divided into small pieces and then subcutaneously inoculated on both flank of another mice. For knockdown FTO mice model, two weeks after inoculation, the shFTO#3 lenti-virus injected into the tumor for three consecutive days. For combined medication mice model, intraperitoneal injection of Rhein and Olaparib was started one week after inoculation. Tumor growth was recorded by measurement of two perpendicular diameters using the formula (tumor volume = $(\text{length} \times \text{width}^2)/2$).

23. Ethics approval and consent to participate

This study was carried out in accordance with the recommendations of Requirements

of the Ethical Review System of Biomedical Research Involving Human by National Health and Family Planning Commission of China, Nankai University and Tianjin Union Medical Center Ethics Committee with written informed consent from all subjects. All subjects gave written informed consent in accordance with the Declaration of Helsinki. Approval of use of mice and designed experiments was given by the Laboratory Animal Ethics Committee Nankai University.

24. Statistical analysis

Data were analyzed and mapped with GraphPad Prism 8, and were presented as mean ± SD as indicated. Two-tailed Student's t-test was used to compare means between groups as indicated. One-way ANOVA was used for multiple treatment conditions and $p < 0.05$ was considered significant.

Antibodies used in this study were listed in Supplementary Information: Table S8.

Supplementary Figure Legends

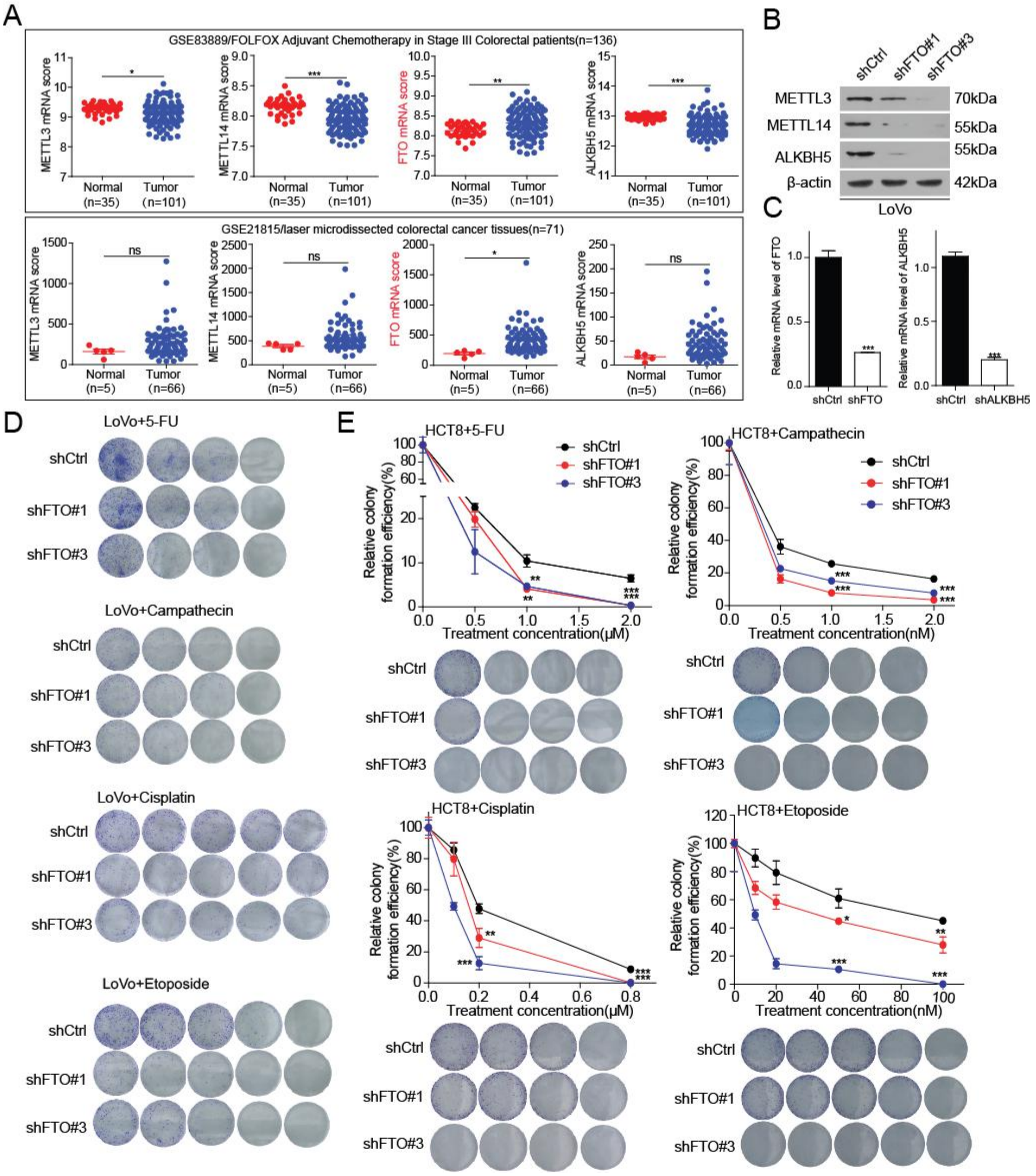

Figure S1. FTO mediated chemotherapy process in CRC. (A) The differential expression of METTL3, METTL14, FTO and ALKBH5 in CRC patients after chemotherapy or laser microdissected was analyzed based on GEO platform. (B) Western blotting analysis of METTL3, METTL14 and ALKBH5 in FTO knockdown cells. (C) Confirmation of FTO and ALKBH5 knockdown in LoVo cells by qPCR. (D-E) The sensitivity of 5-FU, campathecin, cisplatin, and etoposide in FTO knockdown cells. Differences were considered significant when $p < 0.05$ (*), $p < 0.01$ (**) or $p < 0.001$ (***).

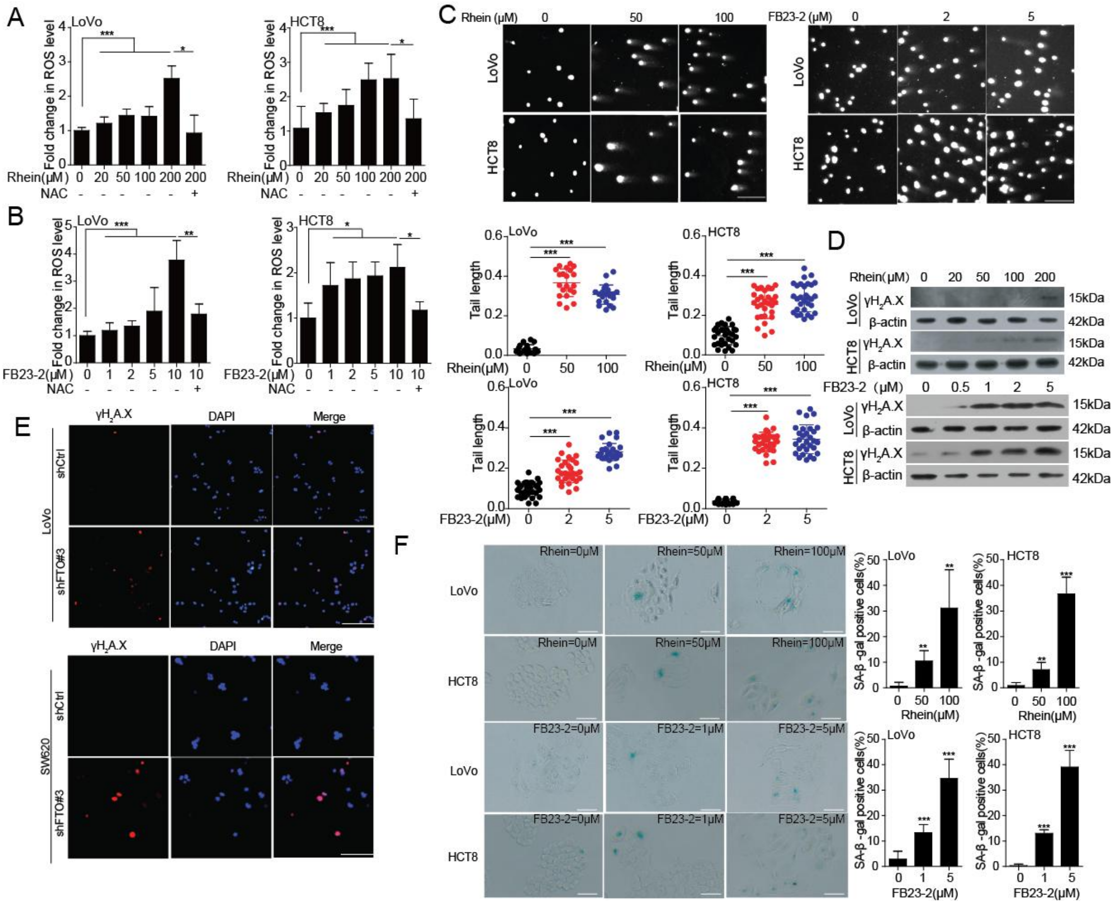

Figure S2. FTO regulates ROS level, DNA damage repair and cell senescence. (A-F) The effect of FTO inhibition on ROS, DNA damage repair and cell senescence. Differences were considered significant when $p < 0.05$ (*), $p < 0.01$ (**) or $p < 0.001$ (***).

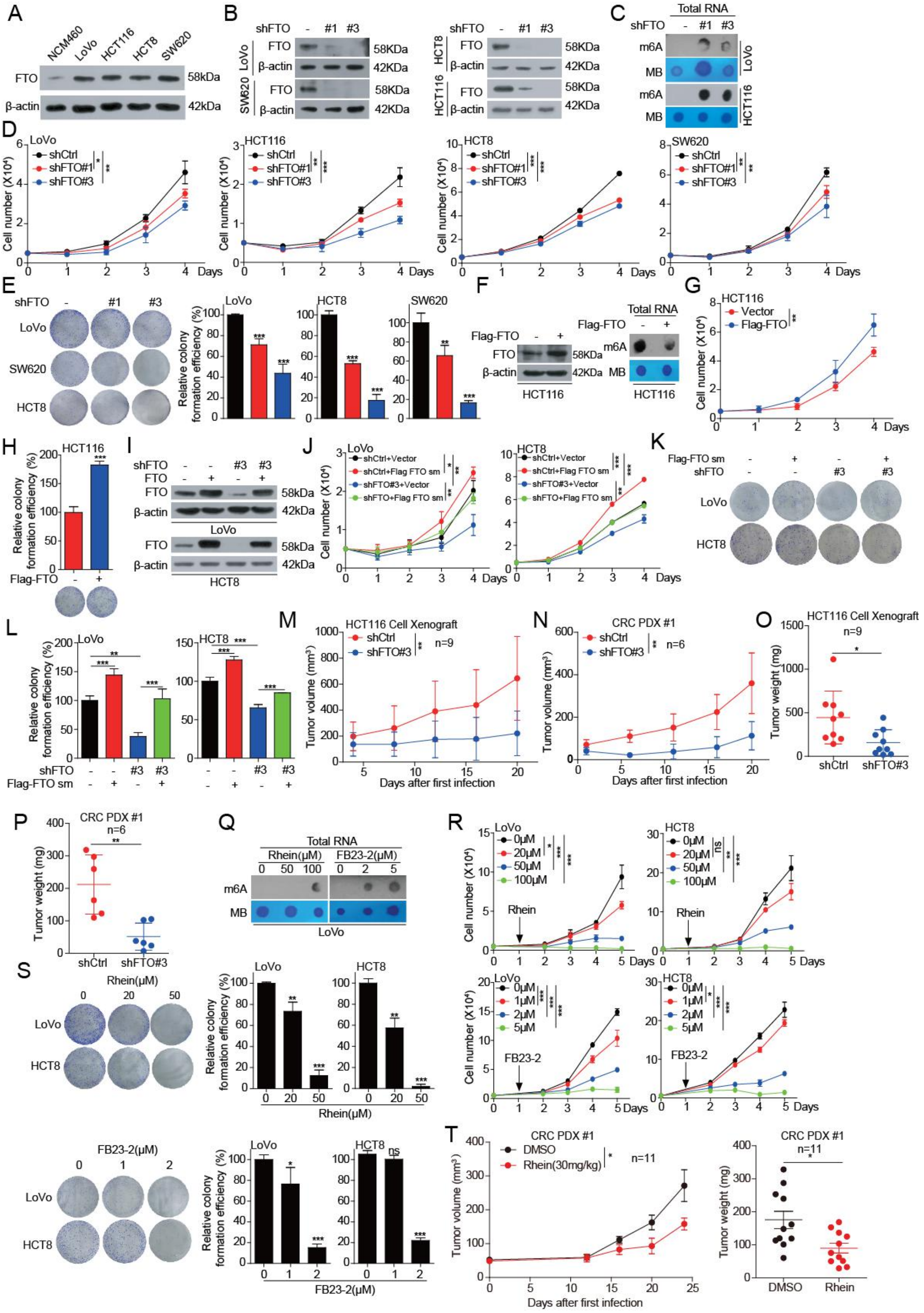

Figure S3. FTO expression is evaluated and promotes CRC cell proliferation and tumor growth. (A) Western blotting analysis of FTO expression in different CRC cells and normal colon epidermal cell. (B) Confirmation of FTO knockdown in LoVo, SW620, HCT8 and HCT116 cells by western blotting analysis. (C) The m6A modification level was determined by dot blotting in LoVo and HCT116 cells with knockdown of FTO. (D) Cell proliferation was determined by cell number counting

assay in FTO stable knockdown cells. (E) Colony formation was determined in FTO stable knockdown cells. (F-H) The cell proliferation and m6A level were determined in exogenous expression of FTO HCT116 cells. (I-L) Cell proliferation and colony formation were determined in FTO knockdown CRC cells with or without re-overexpressing FTO. (M-P) The effect of knockdown of FTO on tumor growth. (Q-T) The effect of FTO inhibition on m6A levels, cell growth and tumor growth. Differences were considered significant when $p < 0.05$ (*), $p < 0.01$ (**) or $p < 0.001$ (***).

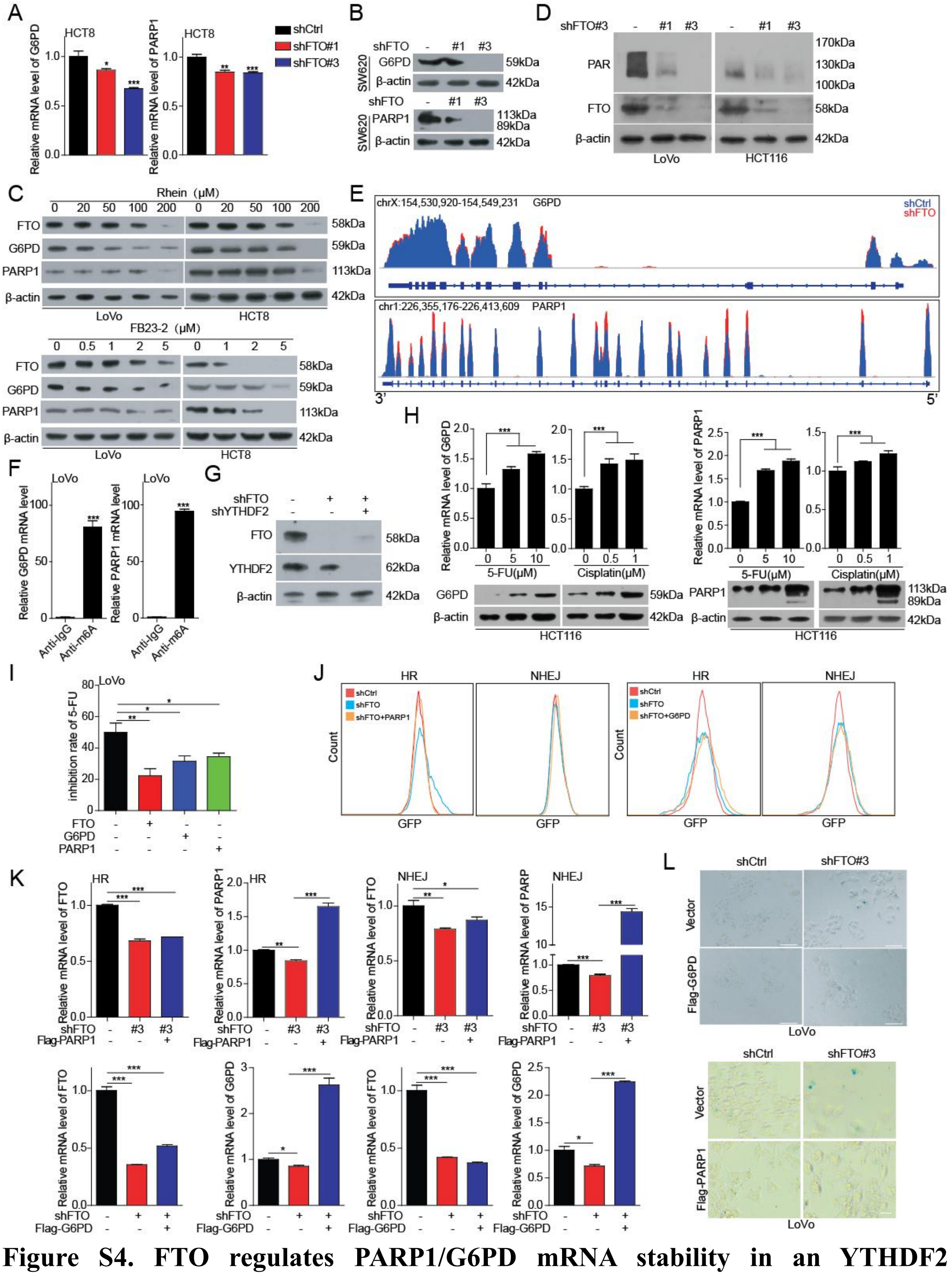

Figure S4. FTO regulates PARP1/G6PD mRNA stability in an YTHDF2

dependent manner. (A-C) The expression of G6PD/PARP1 in FTO knockdown or inhibition cells. (D) The expression of PAR in FTO knockdown cells. (E) The relative abundance of m6A sites along G6PD and PARP1 mRNA in FTO knockdown cells and control cells, as detected by m6A-seq. (F) The MeRIP-qPCR analysis of G6PD and PARP1 m6A levels in LoVo cells. (G) The FTO and YTHDF2 were detected by western blotting in LoVo cells infected with shRNA lenti-virus. (H) The expression of G6PD/PARP1 in CRC cells with treatment of 5-FU or cisplatin. (I) The inhibition rate of 5-FU in LoVo cells expressed FTO/G6PD/PARP1. (J-K) The HR or NHEJ efficiency in FTO knockdown cells with or without overexpression of G6PD/PARP1. The FTO, G6PD and PARP1 were detected by qPCR in U2OS cells infected with shFTO lenti-virus. (L) Restored expression of G6PD/PARP1 antagonizes the cell senescence in FTO knockdown cells. Differences were considered significant when $p < 0.05$ (*), $p < 0.01$ (**) or $p < 0.001$ (***).

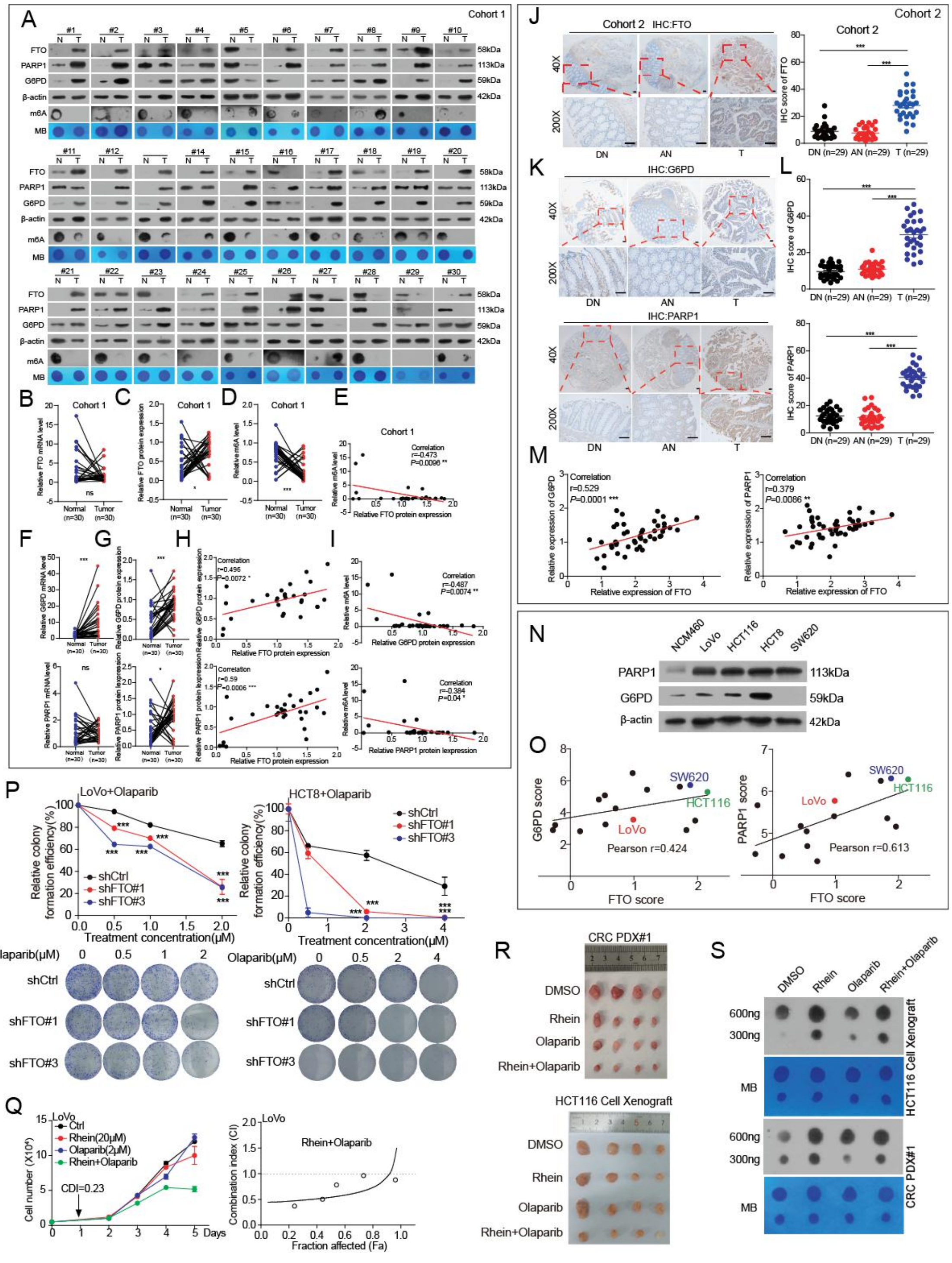

Figure S5. FTO enhanced the anti-tumor effects of Olaparib in CRC. (A-I) Western blotting analysis for FTO, G6PD and PARP1 protein expression and dot blotting for m6A modification level in CRC tissues and paired normal tissues (Cohort 1: n=30). (J-M) Immunohistochemical analysis of FTO, G6PD and PARP1 in a CRC tissues microarray (TMA) containing distant normal tissues (DN), adjacent normal tissues (AN) and CRC tumor tissues (T) (Cohort 2: n=29). Quantification of FTO, G6PD and PARP1 expression by image J software from IHC data. (N) Western blotting analysis of PARP1 and G6PD expression in different CRC cells and normal colon epidermal cell. (O) The correlation between G6PD/PARP1 and FTO expression

in colorectal cancer cells based on CCLE database. (P-Q) Knockdown or inhibition of FTO enhances the sensitivity of CRC to Olaparib. (R) Subcutaneous tumor models in nude mice showed tumor morphology after the treatment of Rhein and Olaparib (n=4 mice per group). (S) The m6A level in mice after the treatment of Rhein and Olaparib. Differences were considered significant when $p < 0.05$ (*), $p < 0.01$ (**) or $p < 0.001$ (***).

Supplementary Tables

Table S1. Expression of FTO protein in colorectal cancer (CRC)

Diagnosis	No of case	FTO			Positive cases rate (%)	Strongly potitive cases rate (%)
		-	+	++		
CRC	29	1	14	14	96.6%***	48.3%***
Adjacent normal colon	29	2	21	6	93.1%	20.7%
Distant normal colon	29	1	20	8	96.6%	27.6%

Positive rate: percentage of positive cases with + and ++ staining score.

Strongly positive rate (high-level expression): percentage of positive cases with ++ staining score.

*** P<0.001 compared with normal colon.

Table S2. Relationship between FTO protein overexpression and the clinic pathological features of CRC

Variables	No. of case	FTO strong positive rate (%)	*P* value
**Gender**			
Male	18	6(33.3%)	0.206
Female	11	6(54.5%)	
**Age (years)**			
>62	13	5(38.5%)	0.405
≤62	16	7(43.8%)	
**Tumor size**			
≤4cm	15	6(40.0%)	0.297
>4cm	14	6(42.9%)	
**Differentiation grade**			
Low and low-middle	8	4(50.0%)	0.493
Middle and high	21	8(38.1%)	
**TNM stage**			
Ia	1	0(0.0%)	0.588
IIIa	22	8(36.4%)	
IIIb	6	4(66.7%)	
**Duke's stage**			
B	0	0(0.0%)	N/A
C	29	12(41.4%)	

Table S3. Expression of G6PD protein in colorectal cancer (CRC)

Diagnosis	No of case	G6PD			Positive cases rate (%)	Strongly potitive cases rate (%)
		-	+	++		
CRC	29	0	8	21	100%***	72.4%***
Adjacent normal colon	29	5	15	9	82.8%	32.4%
Distant normal colon	29	1	19	9	96.6%	31.0%

Positive rate: percentage of positive cases with + and ++ staining score.

Strongly positive rate (high-level expression): percentage of positive cases with ++ staining score.

*** *P*<0.001 compared with normal colon.

Table S4. Relationship between G6PD protein overexpression and the clinic pathological features of CRC

Variables	No. of case	G6PD strong positive rate (%)	*P* value
**Gender**			
Male	18	8(44.4%)	0.427
Female	11	3(27.3%)	
**Age (years)**			
>62	13	6(46.2%)	0.201
≤62	16	5(31.3%)	
**Tumor size**			
≤4cm	15	6(40.0%)	0.944
>4cm	14	5(35.7%)	
**Differentiation grade**			
Low and low-middle	8	4(50.0%)	0.746
Middle and high	21	7(33.3%)	
**TNM stage**			
Ia	1	0(0.0%)	0.102
IIIa	22	6(27.3%)	
IIIb	6	5(83.3%)	
**Duke's stage**			
B	0	0(0.0%)	N/A
C	29	11(37.9%)	

Table S5. Expression of PARP1 protein in colorectal cancer (CRC)

Diagnosis	No of case	PARP1			Positive cases rate (%)	Strongly potitive cases rate (%)
		-	+	++		
CRC	29	0	11	18	100%***	62.1%***
Adjacent normal colon	29	4	22	3	86.2%	10.3%
Distant normal colon	29	1	24	4	96.6%	13.8%

Positive rate: percentage of positive cases with + and ++ staining score.

Strongly positive rate (high-level expression): percentage of positive cases with ++ staining score.

*** P<0.001 compared with normal colon.

Table S6. Relationship between PARP1 protein overexpression and the clinic pathological features of CRC

Variables	No. of case	PARP1 strong positive rate (%)	*P* value
**Gender**			
Male	18	5(27.8%)	0.461
Female	11	4(36.4%)	
**Age (years)**			
>62	13	4(30.8%)	0.605
≤62	16	5(31.3%)	
**Tumor size**			
≤4cm	15	6(40.0%)	0.292
>4cm	14	3(21.4%)	
**Differentiation grade**			
Low and low-middle	8	2(25.0%)	0.311
Middle and high	21	7(33.3%)	
**TNM stage**			
Ia	1	0(0.0%)	0.56
IIIa	22	7(31.8%)	
IIIb	6	2(33.3%)	
**Duke's stage**			
B	0	0(0.0%)	N/A
C	29	9(31.0%)	

Table S7. RT-PCR primers used in this study

Name	Forward	Reverse
FTO	ACTTGGCTCCCT TATCTGACC	TGTGCAGTGTGAGAAAGGCTT
G6PD	CGAGGCCGTCACCAAGAAC	CGAGGCCGTCACCAAGAAC
PARP1	CGGAGTCTTCGGATAAGCTCT	TTTCCATCAAACATGGGCGAC
ALKBH5	CGGCGAAGGCTACACTTACG	CCACCAGCTTTTGGATCACCA
YTHDF2	CCTTAGGTGGAGCCATGATTG	TCTGTGCTACCCAACTTCAGT
PARP1site1	GGAGGAGCAGCTGTGGATCCTG	TCCACCAGGCCAAGGGTGGC
PARP1site2	CTGACATTAAGGTGGTTGACAGAG	CGATGACTTCCAAGTCATACGC
PARP1site3	CGCCTGAAGCGCCCGTGA	GGTCTCCCTGAGACGTATGGC
PARP1site4	CTGACATAGAGAAAAGGCTGGAG	CCCTGGGGAAACCAGTAAGG
G6PDsite1	GTTCCCGCCTCACAGTGGC	CATCGTACTGGCCAGCCACAT
G6PDsite2	GGTGAACCCCCACAAGCTCTG	GCAGCTGAGGTCAATGGTCCC

Table S8. Antibodies for immunoblotting, immunoprecipitation, immunohistochemistry and immunofluorescence in this study.

Primary antibodies	Dilution	Company/Catalog
FTO	WB (1:1000), IHC (1:150)	Proteintech/27226-1-AP
PARP1	WB (1:5000)	Proteintech/66520-1-Ig
PARP1	IHC (1:200)	Proteintech/13371-1-AP
G6PD	WB (1:5000), IHC (1:200)	Proteintech/25413-1-AP
YTHDF2	WB (1:2000)	Proteintech/24744-1-AP
FLAG	WB (1:3000)	Proteintech/20543-1-AP
Ki67	WB (1:1500)	Proteintech/27309-1-AP
β-actin	WB (1:5000)	Proteintech/66009-1-Ig
m6A	DB (1:1000)	Abcam/ab151230
m6A	DB (1:1000)	Abcam/ab190886
$\gamma H_2A.X$	WB (1:1000)	Millipore/05-636
$\gamma H_2A.X$	WB (1:1000)	CST/971
